# Correction: Aqueous spice extracts as alternative antimycotics to control highly drug resistant extensive biofilm forming clinical isolates of *Candida albicans*

**DOI:** 10.1371/journal.pone.0318383

**Published:** 2025-01-24

**Authors:** Bindu Sadanandan, Vaniyamparambath Vijayalakshmi, Priya Ashrit, Uddagiri Venkanna Babu, Lakavalli Mohan Sharath Kumar, Vasulingam Sampath, Kalidas Shetty, Amruta Purushottam Joglekar, Rashmi Awaknavar

Fig 13 is uploaded incorrectly. Please see the correct Fig 13 here.

**Fig 13:**
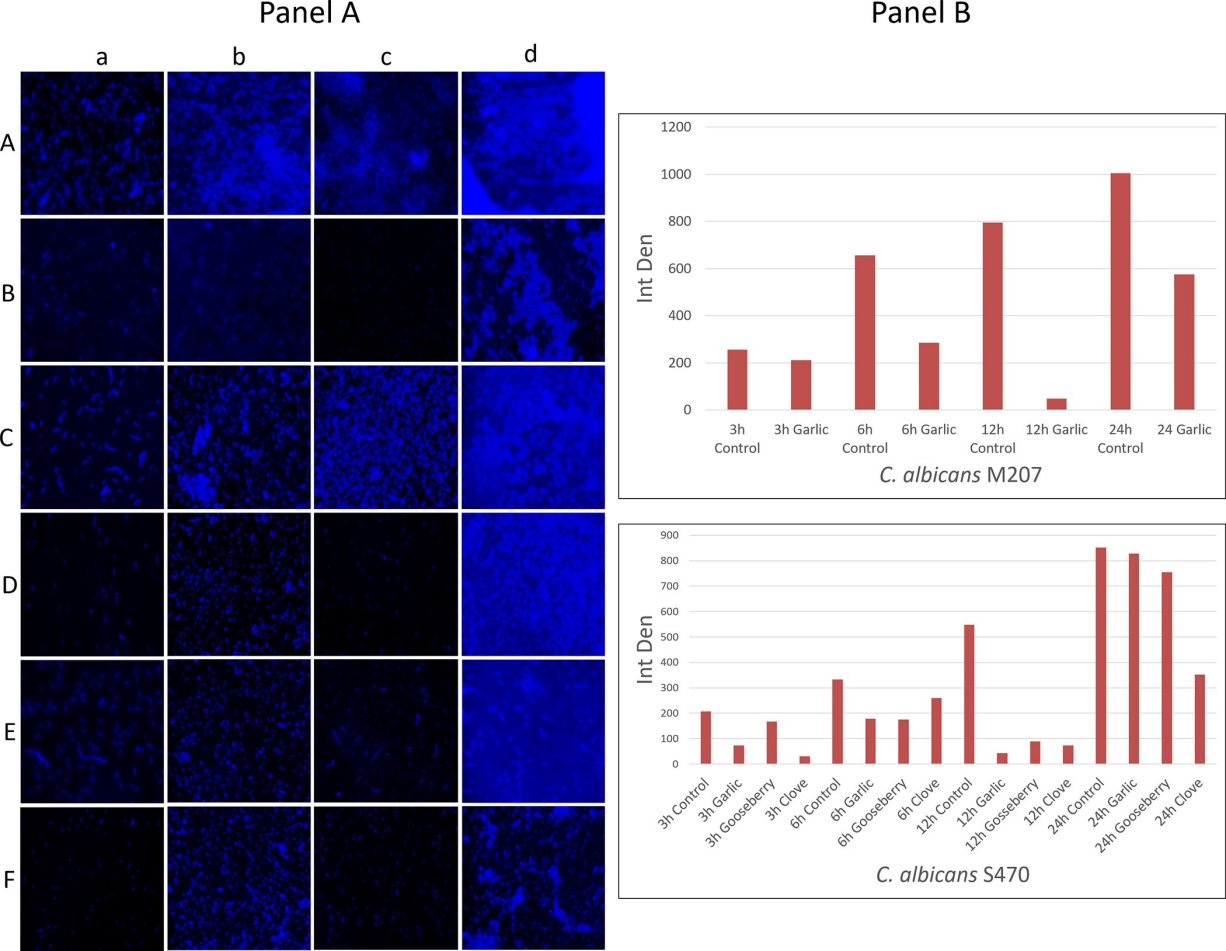
Fluorescence images of *C*. *albicans* stained with calcofluor white. Panel A: Images captured at different time intervals (a) 3 h, (b) 6 h, (c) 12 h, (d) 24 h for (A) *C*. *albicans* M-207 control, (B) Aqueous garlic treated *C*. *albicans* M-207 (1mg), (C) *C*. *albicans* S-470 control, (D) Aqueous garlic treated *C*. *albicans* S-470 (1mg), (E) Aqueous clove treated *C*. *albicans* S-470 (0.215mg), (F) Aqueous Indian gooseberry treated *C*. *albicans* S-470 (0.537mg). All images were captured at 40x magnification. Concentrations are expressed as dry weight measurements. Panel B: Quantification of fluorescence intensity of *C*. *albicans* M-207 and S-470 treated with garlic, clove, and gooseberry at different time intervals analyzed by the fluorescence of the entire image using ImageJ.
