# Supplementary figures and images for: Aqueous spice extracts as alternative antimycotics to control highly drug resistant extensive biofilm forming clinical isolates of Candida albicans
Source: PLoS One. 2023 Jun 14;18(6):e0281035. doi: 10.1371/journal.pone.0281035 (PMC10266687; doi:10.1371/journal.pone.0281035)

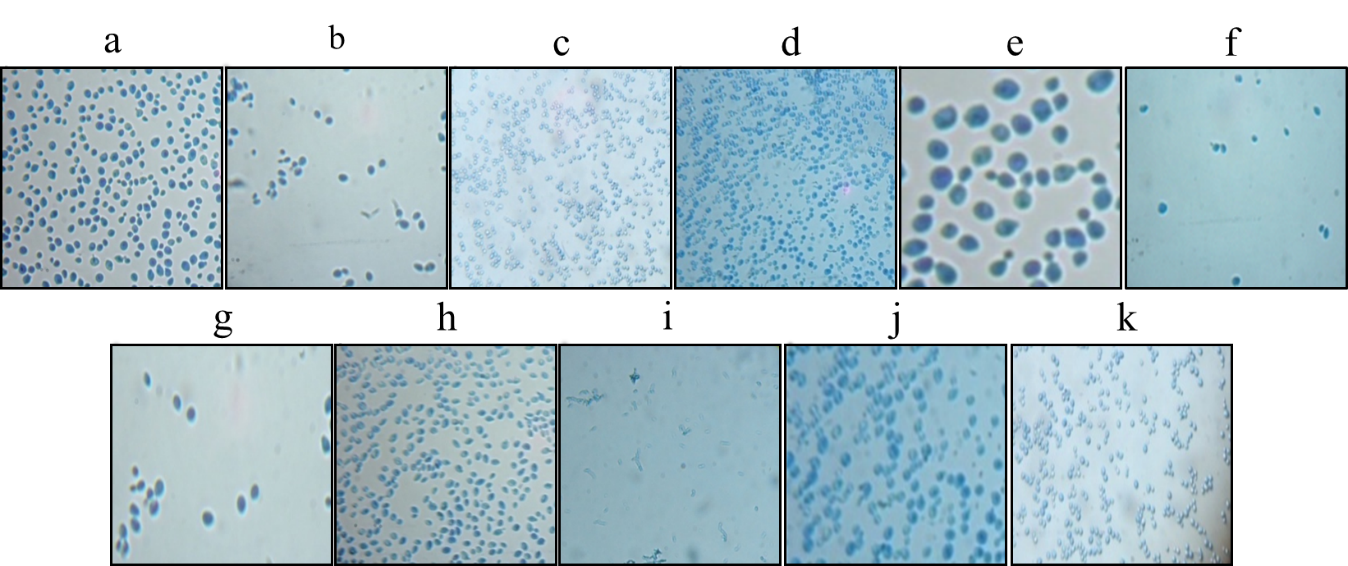

Supplement: S1 Fig — (a) MTCC-3017, (b) U-427, (c) U-499, (d) U-3893, (e) M-207, (f) M-529, (g) S-470, (h) U-2647, (i) U-3800, (j) U-3713, (k) D4 at 24 h. (TIF) [file pone.0281035.s001.tif]

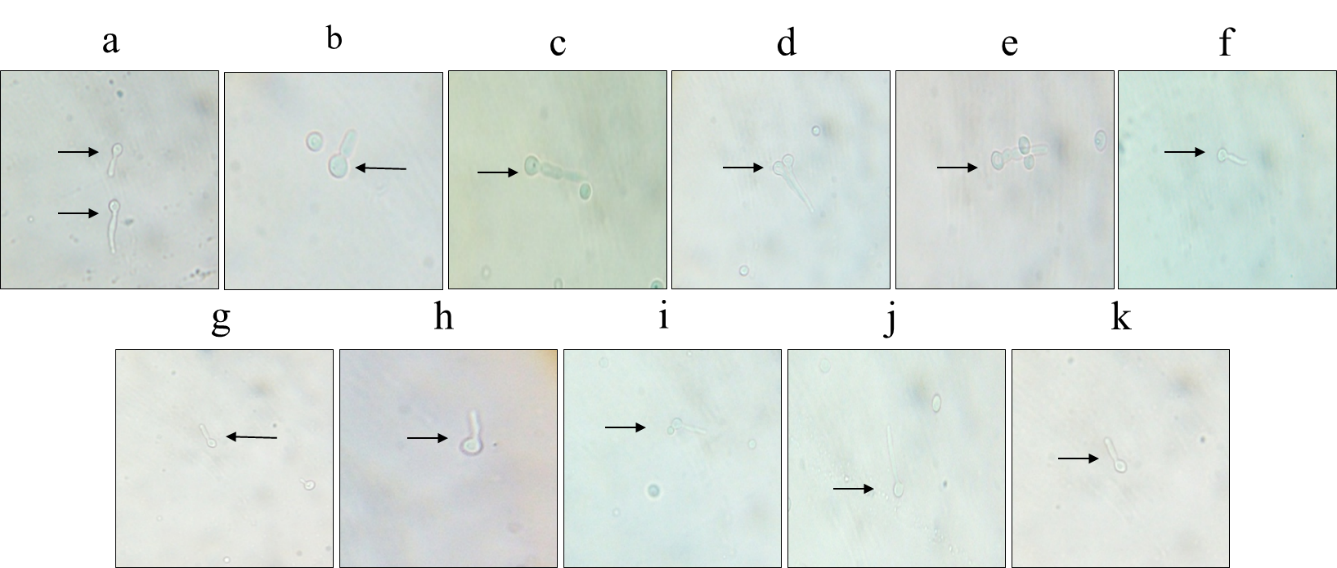

Supplement: S2 Fig — (a) MTCC-3017, (b) M-207, (c) M-529, (d) S-470, (e) U-2647, (f) U-3713, (g) U-3800, (h) U-3893, (i) U-427, (j) U-499, and (k) D-4. The arrows indicate the germ tube formed by the isolates. (TIF) [file pone.0281035.s002.tif]

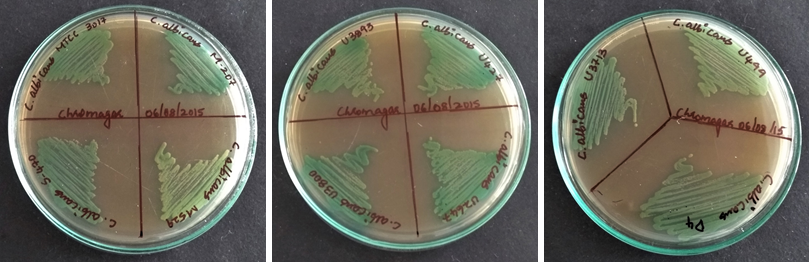

Supplement: S3 Fig — MTCC-3017, M-207, M-529, S-470, U-2647, U-3713, U-3800, U-3893, U-427, U-499, and D-4 were cultured. (TIF) [file pone.0281035.s003.tif]

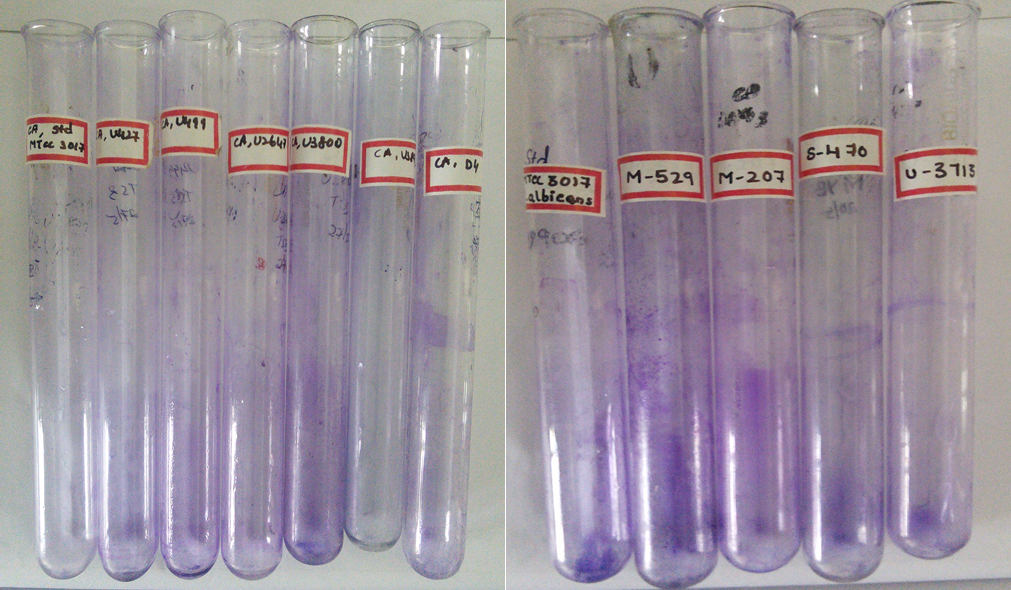

Supplement: S4 Fig — MTCC-3017, M-207, M-529, S-470, U-2647, U-3713, U-3800, U-3893, U-427, U-499, and D-4. (TIF) [file pone.0281035.s004.tif]

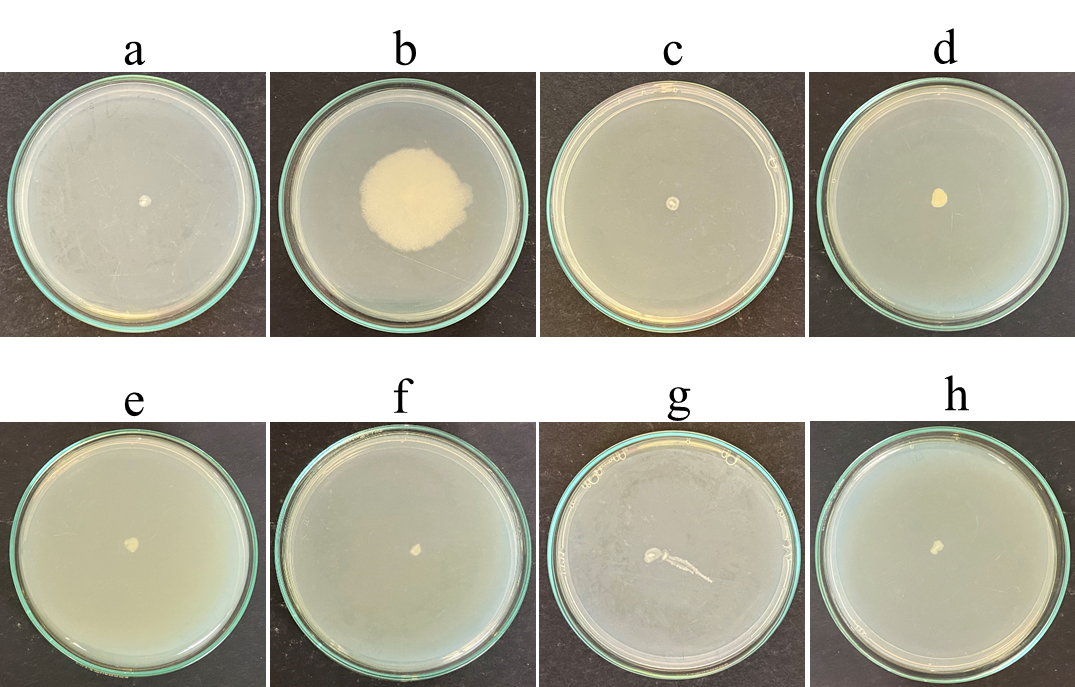

Supplement: S5 Fig — (a) U-427, (b) U-499, (c) U-2647, (d) U-3800, (e) U-3893, (f) U-3713, (g) M-529, and (h) D-4 at 16 h. (TIF) [file pone.0281035.s005.tif]

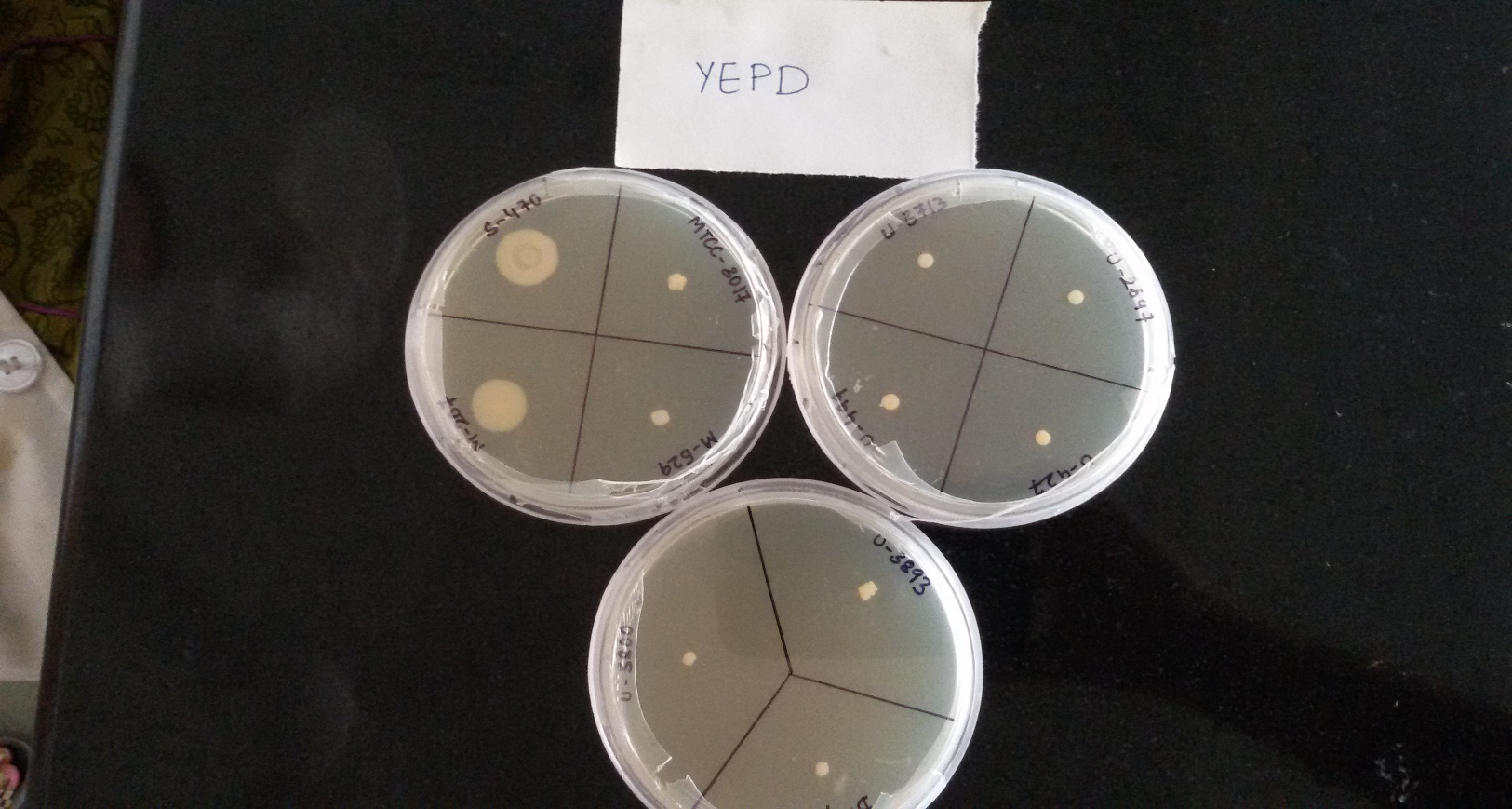

Supplement: S6 Fig — MTCC-3017, M-207, M-529, S-470, U-2647, U-3713, U-3800, U-3893, U-427, U-499, and D-4 at 16 h. (TIF) [file pone.0281035.s006.tif]

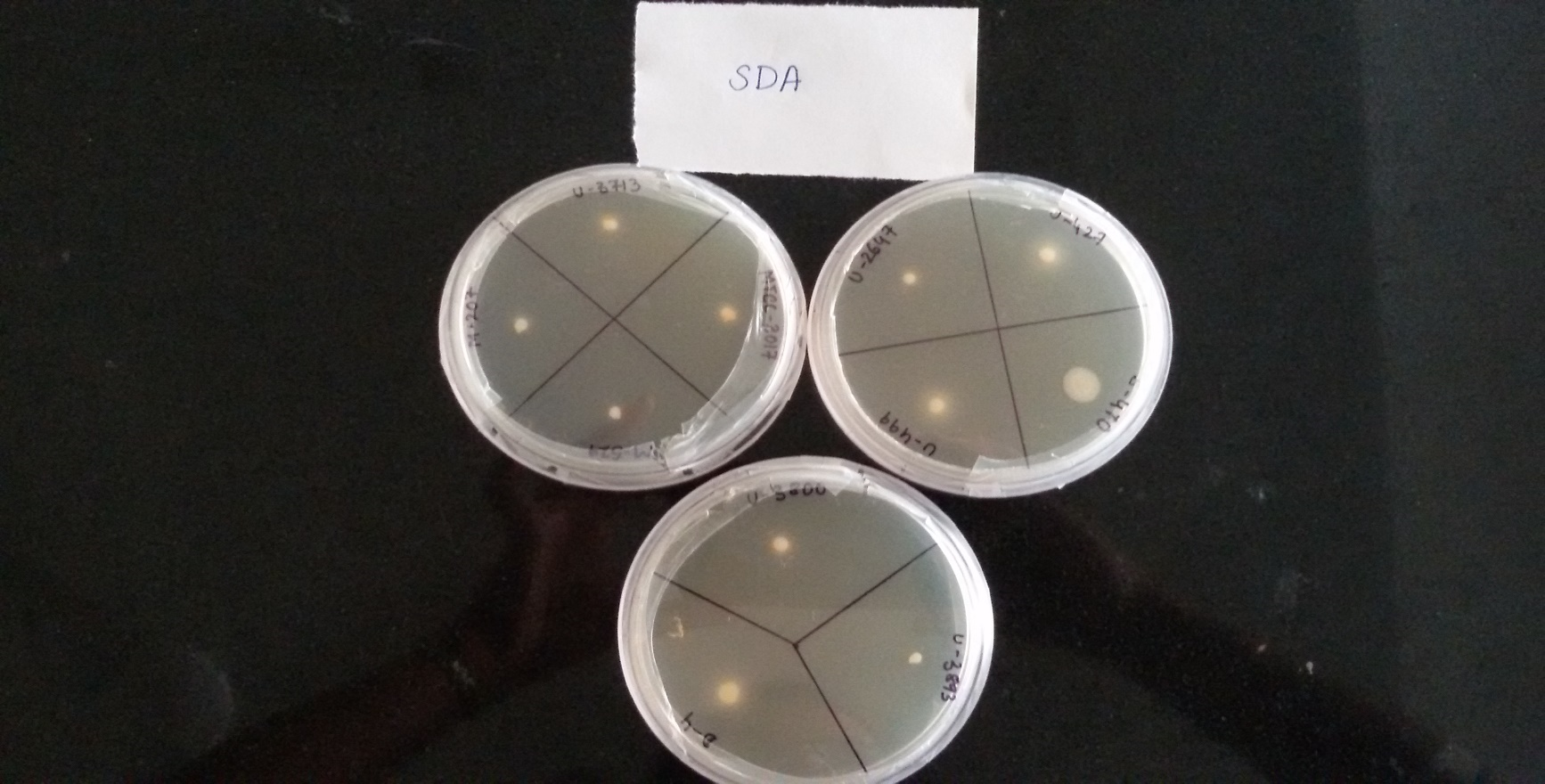

Supplement: S7 Fig — MTCC-3017, M-207, M-529, S-470, U-2647, U-3713, U-3800, U-3893, U-427, U-499, and D-4 at 16 h. (TIF) [file pone.0281035.s007.tif]

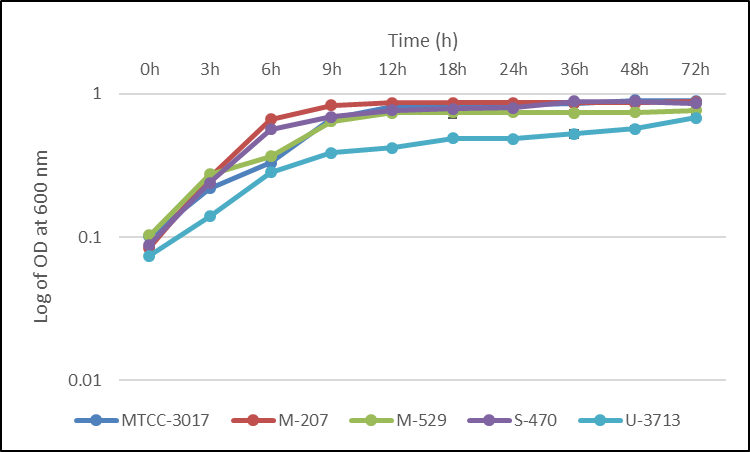

Supplement: S14 Fig — All values are expressed as mean and standard deviation. The experiment was performed in triplicate. (TIF) [file pone.0281035.s014.tif]

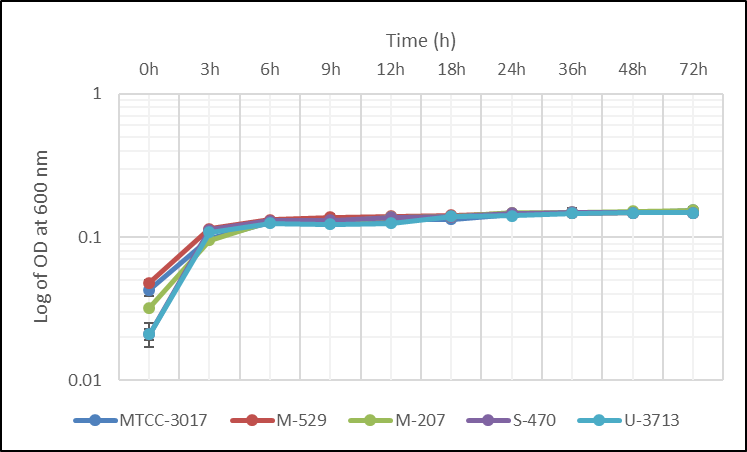

Supplement: S15 Fig — All values are expressed as mean and standard deviation. The experiment was performed in triplicate. (TIF) [file pone.0281035.s015.tif]

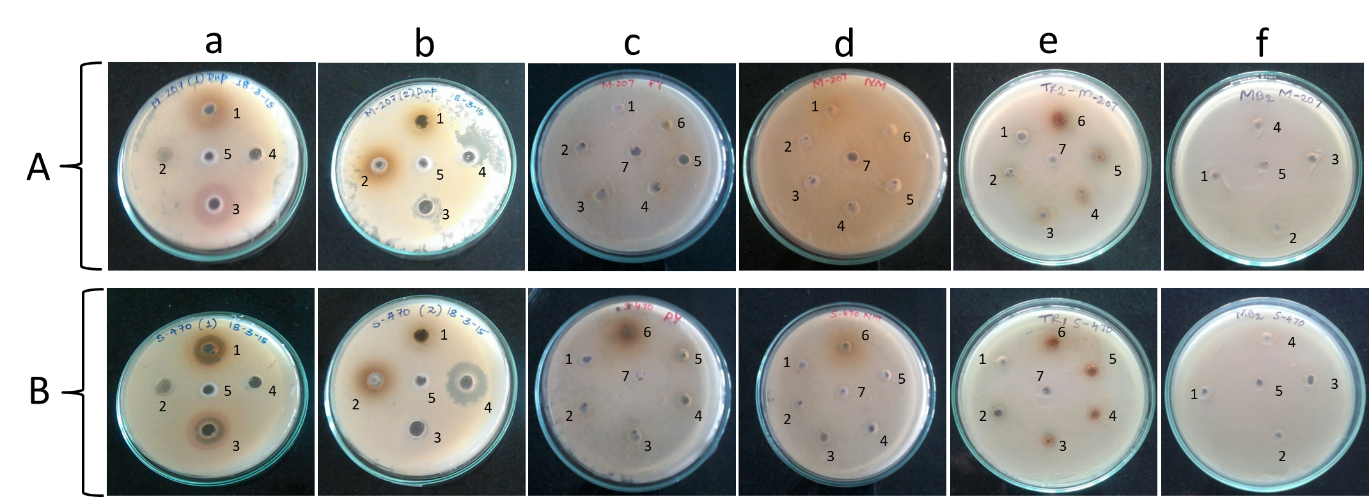

Supplement: S16 Fig — (a) (1) Clove (2) Papaya seeds (3) Indian Gooseberry (4) Onion (5) Water as control, (b) (1) Pudina (2) Pepper (3) Lemon (4) Garlic (5) Water as control, (c) Papaya leaf (1) 100 mgmL-1 (2) 250 mgmL-1 (3) 500 mgmL-1 (4) 750 mgmL-1 (5) 1000 mgmL-1 (6) 2000 mgmL-1 (7) Water as control, (d) Neem (1) 100 mgmL-1 (2) 250 mgmL-1 (3) 500 mgmL-1 (4) 750 mgmL-1 (5) 1000 mgmL-1 (6) 2000 mgmL-1 (7) Water as control, (e) Turmeric (1) 100 mgmL-1 (2) 250 mgmL-1 (3) 500 mgmL-1 (4) 750 mgmL-1 (5) 1000 mgmL-1 (6) 2000 mgmL-1 (7) Water as control, (f) Swallow root (1) 100 mgmL-1 (2) 150 mgmL-1 (3) 250 mgmL-1 (4) 500 mgmL-1 (5) Water as control. (A) C. albicans M-207 and (B) C. albicans S-470 biofilm. (TIF) [file pone.0281035.s016.tif]

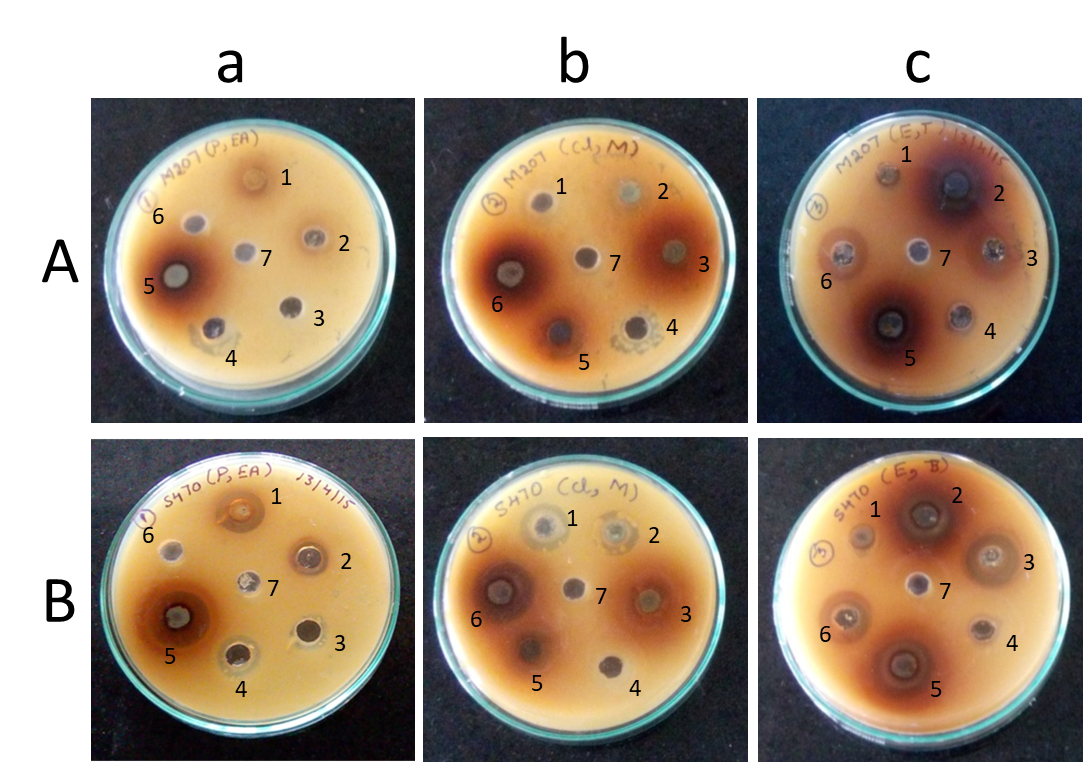

Supplement: S17 Fig — (A) C. albicans M-207 and (B) C. albicans S-470 by solvent extraction. (a) (1) Clove + Petroleum ether, (2) Clove + Ethyl Acetate, (3) Garlic + Petroleum ether, (4) Garlic + Ethyl Acetate, (5) Indian Gooseberry + Ethyl Acetate, (6) Indian Gooseberry + Petroleum ether, (7) Control (Water), (b) (1) Garlic + Chloroform, (2) Indian Gooseberry + Chloroform, (3) Clove + Chloroform, (4) Garlic + Methanol, (5) Indian Gooseberry + Methanol, (6) Clove + Methanol, (7) Control (Water) (c) (1) Garlic + Ethanol, (2) Indian Gooseberry + Ethanol, (3) Clove + Ethanol, (4) Garlic + Butanol, (5) Indian Gooseberry + Butanol, (6) Clove + Butanol, (7) Control (Water). (TIF) [file pone.0281035.s017.tif]

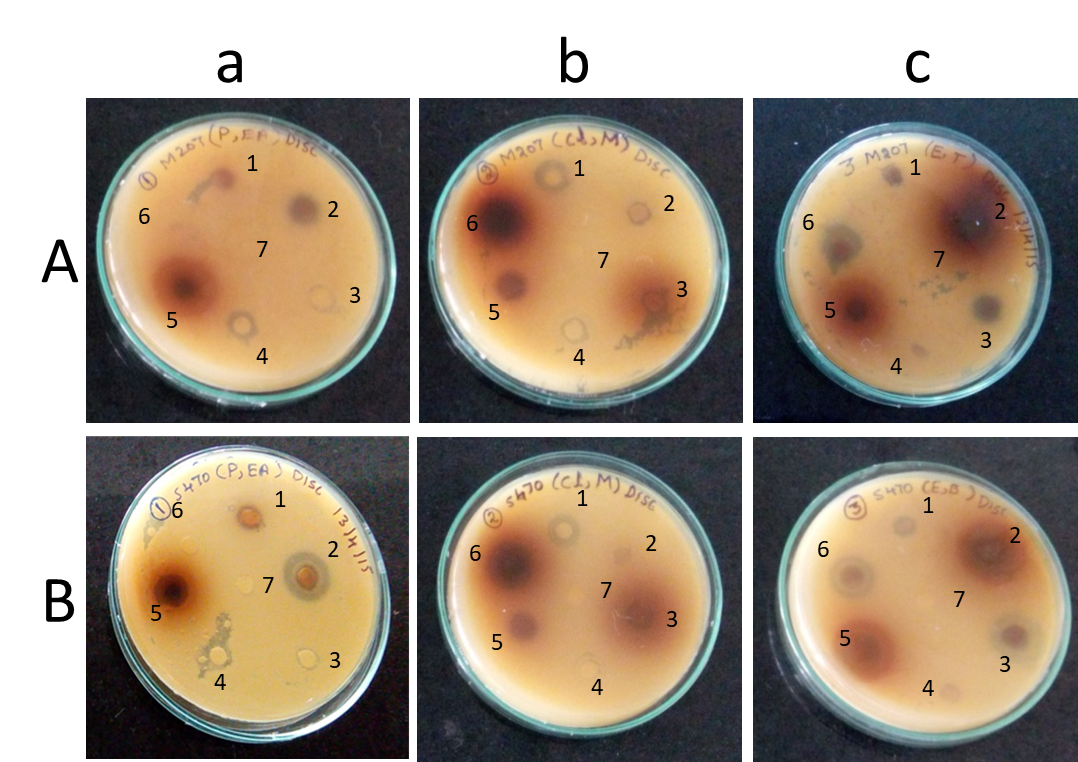

Supplement: S18 Fig — (A) C. albicans M-207 and (B) C. albicans S-470 by solvent extraction. (a) (1) Clove + Petroleum ether, (2) Clove + Ethyl Acetate, (3) Garlic + Petroleum ether, (4) Garlic + Ethyl Acetate, (5) Indian Gooseberry + Ethyl Acetate, (6) Indian Gooseberry + Petroleum ether, (7) Control (Water), (b) (1) Garlic + Chloroform, (2) Indian Gooseberry + Chloroform, (3) Clove + Chloroform, (4) Garlic + Methanol, (5) Indian Gooseberry + Methanol, (6) Clove + Methanol, (7) Control (Water (c) (1) Garlic + Ethanol, (2) Indian Gooseberry + Ethanol, (3) Clove + Ethanol,(4) Garlic + Butanol, (5) Indian Gooseberry + Butanol, (6) Clove + Butanol, (7) Control (Water). (TIF) [file pone.0281035.s018.tif]

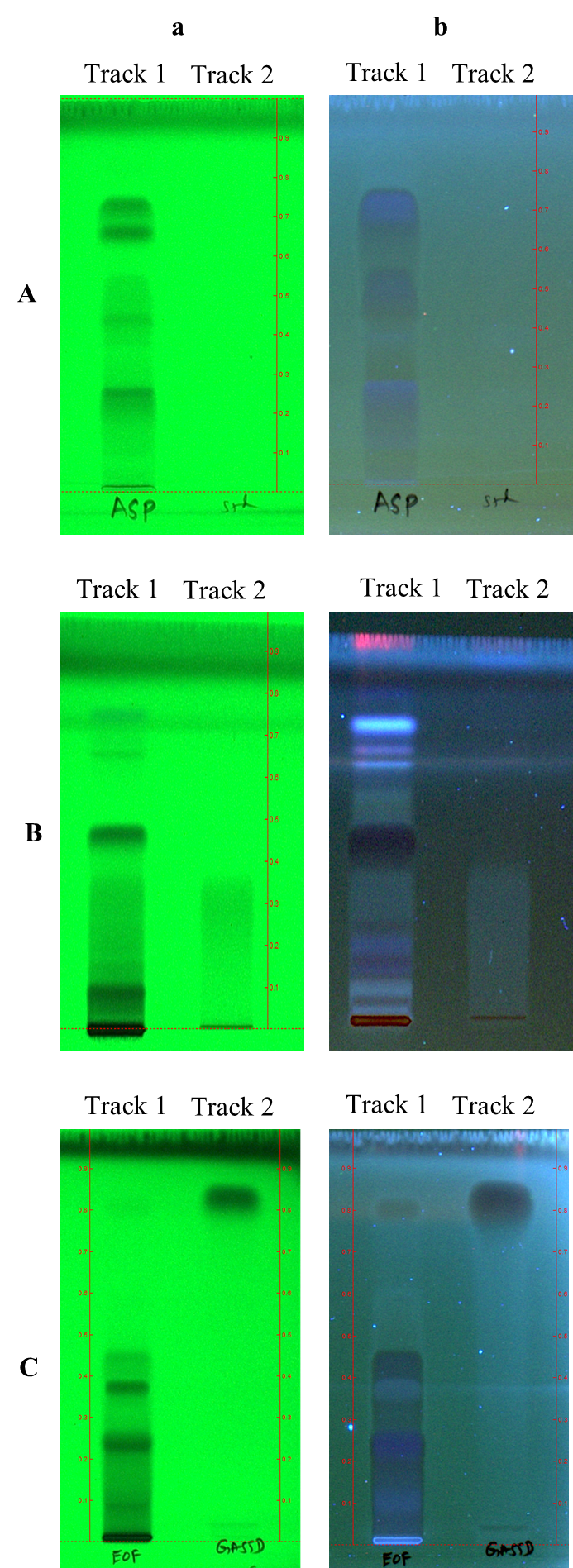

Supplement: S19 Fig — (A) HPTLC of Aqueous garlic extract indicated as ASP (Allium sativum Pulp)—Track 1: ASP (Aq. extract); Track 2: Alliin standard, (B) HPTLC of Aqueous clove extract indicated as SAFB (Syzygium aromaticum Flower Bud)—Track 1: SAFB; Track 2: Ellagic acid standard, (C) HPTLC of Aqueous gooseberry extract indicated as EOF (Emblica officinalis Fruit)—Track 1: EOF; Track 2: Gallic acid standard, at (a) 254 nm and (b) 366 nm. (TIF) [file pone.0281035.s019.tif]

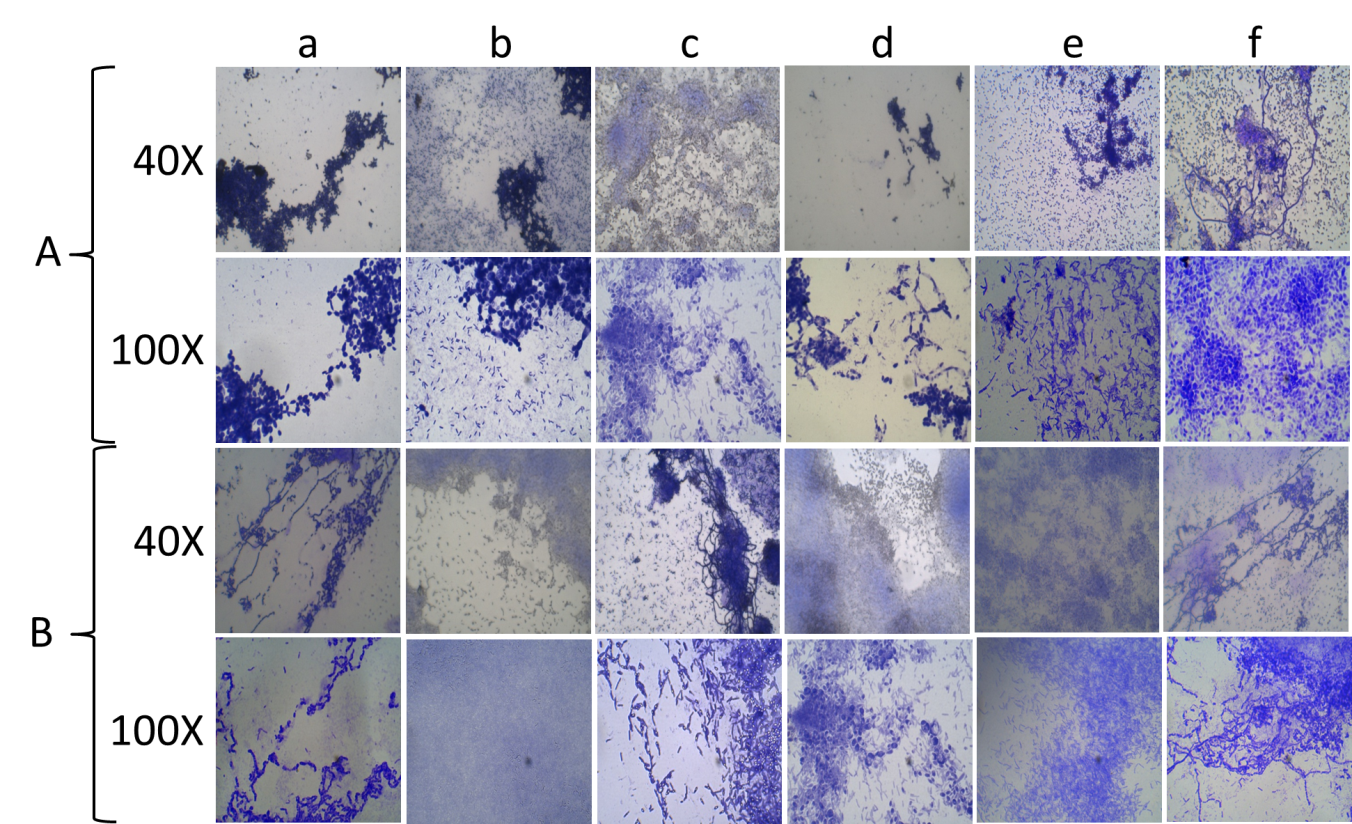

Supplement: S20 Fig — (A) C. albicans M-207, (B) C. albicans S-470 induced on coverslip in TSB medium for (a) 24h, (b) 48h, (c) 72h, (d) 96h, (e) 120h, (f) 12days at 40X & 100X magnifications. (TIF) [file pone.0281035.s020.tif]

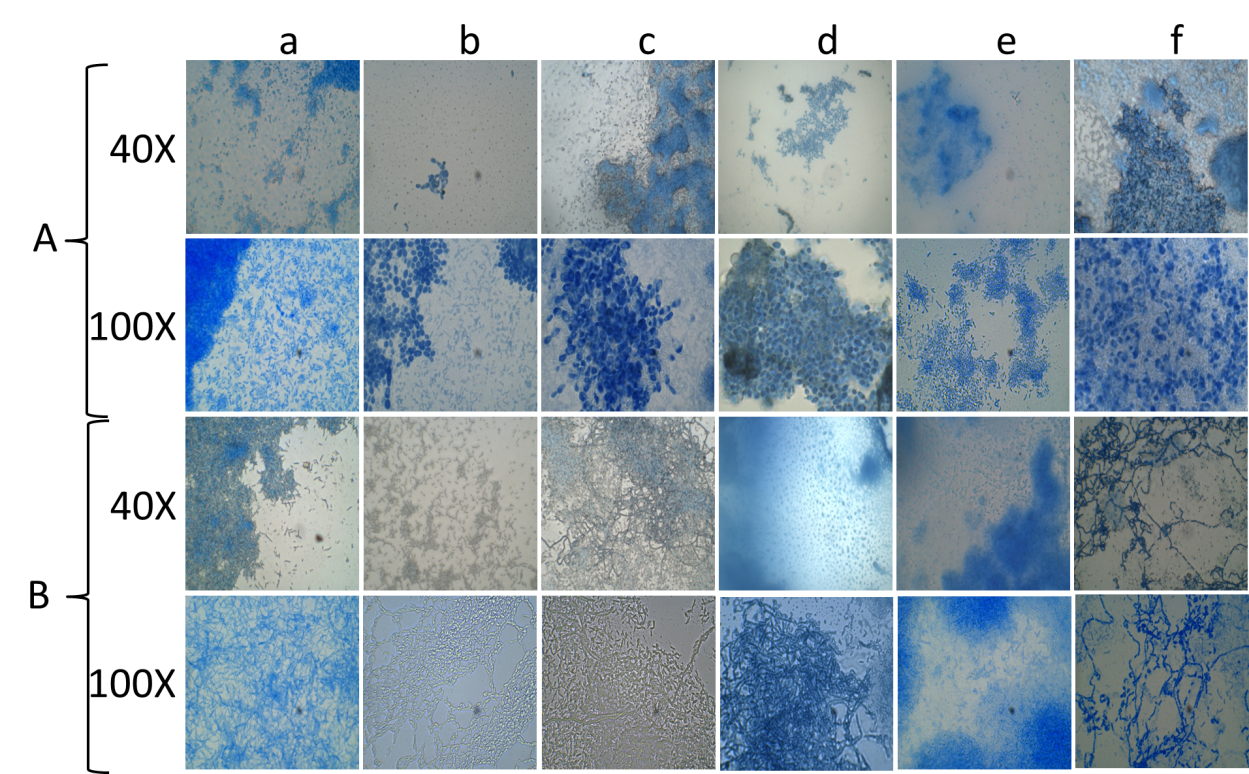

Supplement: S21 Fig — (A) C. albicans M-207, (B) C. albicans S-470 induced on coverslip in TSB medium for (a) 24h, (b) 48h, (c) 72h, (d) 96h, (e) 120h, (f) 12days at 40X & 100X magnifications. (TIF) [file pone.0281035.s021.tif]
