# Supplementary material for: Aqueous spice extracts as alternative antimycotics to control highly drug resistant extensive biofilm forming clinical isolates of Candida albicans
Source: PLoS One. 2023 Jun 14;18(6):e0281035. doi: 10.1371/journal.pone.0281035 (PMC10266687; doi:10.1371/journal.pone.0281035)
Supplement: S1 Table — The solvents used were petroleum ether, ethyl acetate, chloroform, methanol, ethanol, and butanol. (PDF) [file pone.0281035.s022.pdf]

| **Agents/Solvents** | **Zone of Inhibition (mm) for *C. albicans* M-207 (well)** | **Zone of Inhibition (mm) for *C. albicans* M-207 (disc)** | **Zone of Inhibition (mm) for *C. albicans* S-470 (well)** | **Zone of Inhibition (mm) for *C. albicans* S-470 (disc)** |
|---|---|---|---|---|
| **Garlic** | | | | |
| **Petroleum ether** | 5 | 6 | 5 | 4 |
| **Ethyl acetate** | 6 | 5.5 | 6.5 | 5.5 |
| **Chloroform** | 5 | 5.5 | 0.5 | 5.5 |
| **Methanol** | 8.5 | 4.5 | 4.5 | 6 |
| **Ethanol** | 5.5 | 5 | 5.5 | 3.5 |
| **Butanol** | 4.5 | 3.5 | - | - |
| **Clove** | | | | |
| **Petroleum ether** | 4.5 | 5 | 8 | 4.5 |
| **Ethyl acetate** | - | 4 | 6 | 8.5 |
| **Chloroform** | 5.5 | 0.5 | 6 | 5 |
| **Methanol** | - | - | 11 | 9.5 |
| **Ethanol** | - | - | 9.5 | 9 |
| **Butanol** | - | 6.5 | 8.5 | 7.5 |
| **Gooseberry** | | | | |
| **Petroleum ether** | - | - | - | - |
| **Ethyl acetate** | - | - | 10.5 | 6.5 |
| **Chloroform** | 4.5 | 4 | 8 | 3 |
| **Methanol** | 7.5 | 4.5 | 8 | 4 |
| **Ethanol** | - | - | 9 | 8.5 |
| **Butanol** | - | - | 10.5 | 7 |
